# Perioperative neuropsychological assessment reveals dynamic changes in neurocognitive function following brain tumor surgery

**DOI:** 10.1016/j.bas.2025.105907

**Published:** 2025-12-13

**Authors:** Pranjali Ektare, Neha Pai, Kanchi Jain, Pallavi Rane, Vikas Kumar Singh, Prakash M. Shetty, Aliasgar V. Moiyadi

**Affiliations:** a Neurosurgical Oncology Services, Department of Surgical Oncology, Tata Memorial Centre, Mumbai, Maharashtra, India; b Department of Health Sciences, Homi Bhabha National Institute, Mumbai, Maharashtra, India; c Clinical Research Secretariat, ACTREC, Tata Memorial Centre, Mumbai, 400012, India

**Keywords:** Neurocognition, Brain tumor, Gliomas, Postoperative outcome

## Abstract

**Introduction:**

Research has shown that patients with primary brain tumours have baseline neurocognitive deficits.

**Research question:**

In the present study, we sought to explore the impact of surgical intervention as it is not adequately understood.

**Materials and methods:**

Patients with intra-axial tumours undergoing surgery were perioperatively evaluated using a comprehensive neuropsychological battery on domains of Attention and Executive Function, memory, Language, Visuomotor Speed and Visuospatial ability. Assessments were done at baseline and post-operatively around 1 month after the surgery before starting any adjuvant treatment (n = 66).

**Results:**

Excluding memory, all domains showed an increased number of patients with severe deficits post-operatively, though the percentage of patients with overall severe deficits decreased. Memory domain was thought to have the most improvement while visuomotor speed had the highest worsening. Patients who underwent craniotomies under general anaesthesia had significant worsening in the memory domain and had an overall trend for worsening across all domains post-op in comparison to those who underwent awake craniotomies. Visuomotor speed was affected by subtotal resection. Tumour lateralisation to the right influenced performance in the visuospatial domain.

**Conclusion:**

There is significant neurocognitive dysfunction in patients with PBTs in the perioperative period with many dynamic changes in the post-operative performance as compared to the baseline. Awake craniotomy can mitigate some of this decline. Detailed cognitive assessments serially performed over the course of treatment is essential to unearth the evolving changes in neurocognition and customise interventions.

## Highlights

• Neurocognition is very often affected in brain tumours.
• We assessed neurocognition before and after brain tumor surgery in 66 cases.
• Significant dynamic changes in neurocognition were observed (both improvements and worsening).
• Individual domain changes were seen even when overall group assessments appeared unchanged.
• Awake craniotomy had a favourable outcome with lesser cognitive decline postoperatively.

## List of abbreviations

NCF: Neuro Cognitive Function
NPA: Neuropsychological Assessment
AC: Awake Craniotomy
GA: General Anaesthesia
QoL: Quality of Life
PBT: Primary Brain Tumours
GTR: Gross Total Resection
EF: Executive Functions
LGG: Low Grade Glioma
GBM: Glioblastoma
IDH: Isocitrate Dehydrogenase

## 1 Introduction

The incidence of PBTs is relatively low in comparison to all other types of cancers, but their morbidity rates are proportionately high, especially in terms of functional impairments (Ostrom et al., 2014).

Gliomas are by far the commonest malignant primary brain tumor group. The 2021 WHO classification (Louis et al., 2021), divides adult diffuse hemispheric gliomas into 3 molecularly defined categories (astrocytoma IDH mutant, oligodendroglioma 1p19q codeleted, and glioblastoma IDH wild type). The progressive nature of diffuse gliomas makes them challenging to treat. The standard of care for primary brain tumours includes a combination of surgery, radiotherapy (RT) and chemotherapy. Advances in treatment such as breakthroughs in radiation therapy, increased capabilities for radical resections, and awake craniotomies for preservation of function have led to an increase in life expectancy with a better quality of life. Most patients with gliomas exhibit some level of impairment in their neurocognitive functioning (Noll et al., 2020; Sleurs et al., 2022), the nature and severity of which is variable. Neurocognitive function (NCF) outcomes are important in identifying patient care variables such as degree of independence, self-care, occupational capacity, social and emotional well-being and overall Quality of Life (QoL) (Yoshida et al., 2020). NCF has also been shown to have predictive value (Armstrong et al., 2003) when used as an adjunct with diagnostic tools like MRI.

Cognitive deficits occurring at the baseline before any treatments are likely associated with the size, location, laterality, co-existing medications, histopathology and the molecular subtype of tumour, all of which can lead to local and distant cerebral network disruptions, in addition to the cognitive reserve (innate resistance to damage in order to maintain functionality, based on factors like years of education, occupation type, demographic features) (6). Treatment itself can exacerbate pre-existing deficits. While surgical interventions are an essential part of the treatment of gliomas, the percentage of patients experiencing post-op NCF decline can be significant. This may be due to direct injury, oedema, post-operative degradation of functional networks, complications caused by seizures, along with personal variables like stress, anxiety, and fatigue, among others. On the other hand, improvements have also been reported, mainly due to the relief of mass effect, which restores previously disrupted networks (Sleurs et al., 2022).

As surgical methods have advanced with the introduction of tools that allow for more precision, detailed imaging techniques, and the wider use of awake craniotomies to help preserve eloquent areas, there is a growing interest in identifying the impact of neurosurgical interventions on gliomas. While abundant literature exists on the impact of RT on neurocognition, the effects of surgery alone on NCF remain relatively obscure, especially in the Indian population, where the cognitive reserve may be different from other populations. In this paper, we attempt to measure postoperative changes in NCF in the cohort of gliomas in the Indian population and attempt to analyse the effect of various factors which may influence these changes.

## 2 Methods

A retrospective longitudinal repeated measures study was conducted at a tertiary care oncology centre in India. Data was obtained with the approval of the internal ethics committee, in line with the institutional policy. All patients with supratentorial intra-axial tumours who underwent tumour resections and for whom paired data was available preoperatively and postoperatively, but before RT initiation, were included. This was to ensure that the effect of RT on NCF would not confound the outcome. A preoperative neuropsychological assessment (NPA) was done in these patients as part of our routine practice. Patients with severe neurological deficits, pre-existing psychiatric illnesses, and patients who could not read and write were excluded as the administration of the standardised battery was not possible. Table 1 lists the battery and tests used. Post-operatively, these patients were followed up prior to radiation between POD15-30 and detailed NPA was again performed. Relevant clinical, radiological, and histopathological data was retrieved from a prospectively maintained neurosurgical database as well as from the hospital's electronic medical records. Histology was recorded from the routine reports in line with the 2021 WHO classification. IDH molecular status (routinely performed for all diffuse gliomas using immunohistochemistry or sequencing when indicated) was also recorded.

**Table 1:** List of Neuropsychological assessments used in this study.

Cognitive domains	Tests used for assessment
Attention and Executive function	1 Digit Vigilance Test 2 Stroop test 3 Controlled Word Association Test 4 Colour Trails 2
Memory	1 Rey Auditory Verbal Learning Test 2 Rey-Osterrieth Complex Figure Test recall
Visuomotor speed	1 Colour Trails 1 2 Digit Symbol Substitution Test
Visuospatial construction	1 Rey-Osterrieth Complex Figure Test copy 2 Line bisection
Language	1 Picture Naming 2 Action words 3 Picture Description

### 2.1 Neurocognitive assessment

Demographic details and handedness were noted as a part of detailed history for each patient. After an initial screening with the Addenbrooke's Cognitive Examination (ACE-III), a detailed battery for neuropsychological tests was administered (Table 1). Five major domains were tested: Attention and Executive Function (A&EF), Memory (verbal and visual), Language, Visuospatial function and Visuomotor speed with domain-specific tests. We have previously reported our NPA assessment and scoring criteria (Moiyadi et al., 2023).

Z scores were calculated and classified as Normal (z < −1 SD), Mild/Moderate (−1 to −2 SD) and Severe (z < −2 SD) for tests where this was available. Others were graded semi-quantitatively. A domain was considered affected if any one of the tests (Table 1) pertaining to that domain had impairments in performance, and severity was categorized based on the worst test result if more than one test per domain was affected. For analysis, the NCF was represented semi-quantitatively on an ordinal three-point score (Normal = 1/Mild-moderate = 2/Severe = 3) for each of the domains. Overall NCF was adjudged based on deficits in overall cognitive profile of the patient, and deficits in any domain led to an assessment of dysfunction in the overall NCF. In case of multi-domain deficits, the deficit with the highest severity was considered. The post-op change in status of patients was obtained by calculating the difference between the post-op and the pre-op performance and classes were obtained in a range of −2 (improvement) to +2 (worsening) for each domain as well as for overall NCF.

### 2.2 Statistical analysis

Categorical data were presented as counts (and percentages) and continuous data as mean (and standard deviation) or median (and interquartile range) as appropriate. The proportion of affected patients was expressed as a percentage of the total number of patients tested, which was variable per domain.

The following analyses were then performed.

1. At the group level, median scores for NCF (ranging from 1 to 3) were calculated for each domain. Pre- and postoperative median scores were compared using a nonparametric test (Wilcoxon Signed Rank test) and depicted using box and whisker plots.
2. Percentage of severely affected patients pre- and postoperatively were compared using the McNemar test. Deficits were grouped as severe and non-severe (mild-moderate group was combined with normal, because of smaller numbers of the sample and because subtle deficits, i.e., within 2-SD of normative data may not be clinically significant).
3. Domain-level changes were evaluable only in cases where paired assessments in the same domain at both time points were done. The pre and postoperative scores of each patient were compared, and the change in individual patient performance postoperatively was calculated by subtracting the preoperative score from the postoperative one. Positive values (+1, +2) were considered as worsening and negative values (−1, −2) were taken as improvements, whereas 0 was considered as stable NCF. At the individual level, the percentage of patients that worsened and improved was calculated. River plots were also constructed to better understand the flux in the NCF status at the individual level.
4. Factors affecting NCF changes were then evaluated as follows:
  a. At the group level – The median change in NCF score (overall as well as individual domains) was compared for the categorical variables [Type of surgery; Laterality; histology; extent of resection] using the Mann-Whitney *U* test.
  b. Worsening rate of individuals – The proportion of patients with worsening of NCF postoperatively was evaluated for the various covariates [Type of surgery; Laterality; histology; extent of resection] using chi-square/Fischer's exact test statistic. For the continuous variable (lesion size), the mean value in worsened and stable patients was compared using the independent student t-test.

P values of less than 0.05 were considered statistically significant. All statistical analyses were performed with SPSS software (IBM SPSS Statistics for Windows, Version 25.0. Armonk, NY: IBM Corp).

## 3 Results

66 patients undergoing surgery had paired NPA pre- and postoperatively during the study period. More patients underwent AC than GA (67 % vs 33 %, p = 0.007). There were more males in the sample than females (70 % vs 30 %, p = 0.001). 69 % of the total patients had tumours that were larger than 4 cm (p = 0.002), and 75 % of the cases had lower-grade gliomas as compared to GBM (p < 0.001). Most patients were right-handed, and college educated. Preoperatively, speech and motor deficits were clinically seen in 4.5 % and 6.1 % of patients, respectively. Table 2 provides the detailed clinicodemographic data of the cohort. The AC group had far more lower-grade gliomas (79 % vs 21 % of GA cases; p < 0.000), consequently having more patients who tested positive for the IDH gene (68 % vs 32 % from GA cases, p < 0.005). GTR rates were higher in the AC group compared to the GA group (63 % vs 37 %), though this was not statistically significant.

**Table 2:** Clinicodemographic characteristics of the study cohort.

Variables	Level	Frequency
Type of surgery	AC	44 (67 %)
	GA	22 (33 %)
Gender	Male	46 (70 %)
	Female	20 (30 %)
Age (in years) Median (SD)		38 (11 %)
Prior treatment for tumour under evaluation	Yes	17 (26 %)
	No	49 (74 %)
Pre-op Neurological Deficits	Speech	3 (4.5 %)
	Motor	4 (6.1 %)
	Both	2 (3.0 %)
	None	57 (86 %)
Tumour laterality	Left	38 (58 %)
	Right	28 (42 %)
Lobes involved	Frontal	43 (65 %)
	Temporal	31 (47 %)
	Parietal	14 (21 %)
	Occipital	2 (3.0 %)
	Insular	22 (33 %)
Histopathology	Lower grade glioma	48 (75 %)
	Glioblastoma	16 (25 %)
IDH status	Positive	34 (56 %)
	Negative	27 (44 %)
Extent of resection	Gross Total Resection (GTR)	35 (53 %)
	Non-GTR	31 (47 %)
Tumour size	>2 cm	3(4 %)
	2–4 cm	18(26 %)
	>4 cm	49(70 %0
KPS	100 %	9(13 %)
	90 %	32(45 %)
	80 %	26(37 %)
	70 %	3(4 %)
	50 %	1(1 %)

### 3.1 Group-level NCF outcomes

Preoperatively, severe NCF deficits were seen in 76 % of the patients. A-EF (63 %) was the most affected domain, followed by Memory (40 %), while Language was the least affected domain at 26 %. At baseline, 70 % of AC and 86 % of GA patients had severe deficits in their overall NCF.

**Fig. 1:**
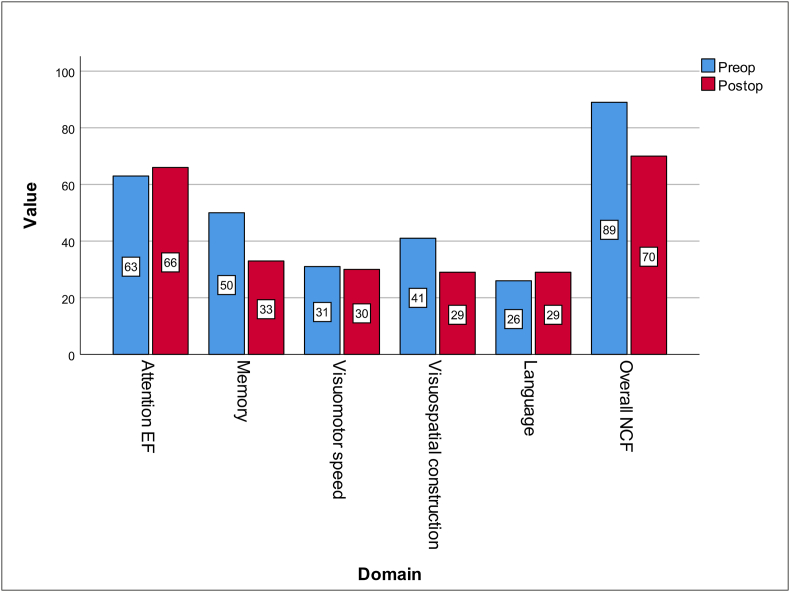
Percentage distribution of severe cognitive deficits pre- and post-operatively visualised by bar diagram of the group scores (preoperative denoted by blue and postoperative scores denoted by red). Note that whereas the overall function seemed to be equally affected in both groups, individual domain functions were variable.

**Fig. 2:**
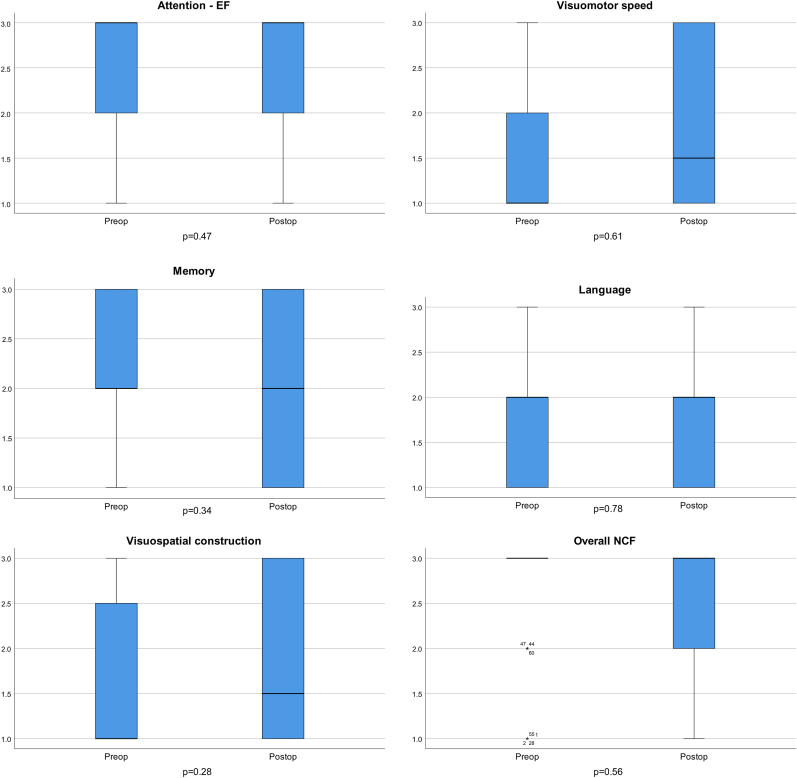
Box and whisker plots depicting group level changes in the neurocognitive function. The boxes depict the central 50th centile distribution (interquartile range) of scores for each domain, and the group median score is depicted by the horizontal bold line within each box, with the whiskers depicting the 25th centiles (quartiles) on either side.

Domains except memory showed an increase in patients with severe deficits in comparison to the pre-op baseline. Interestingly, a decline in the number of patients with severe deficits in overall NCF was seen as the percentage dropped from 76 % to 70 % (Fig. 1). These group-level differences were not found to be statistically significant (Fig. 2).

### 3.2 Individual trends in postoperative NCF

**Fig. 3:**
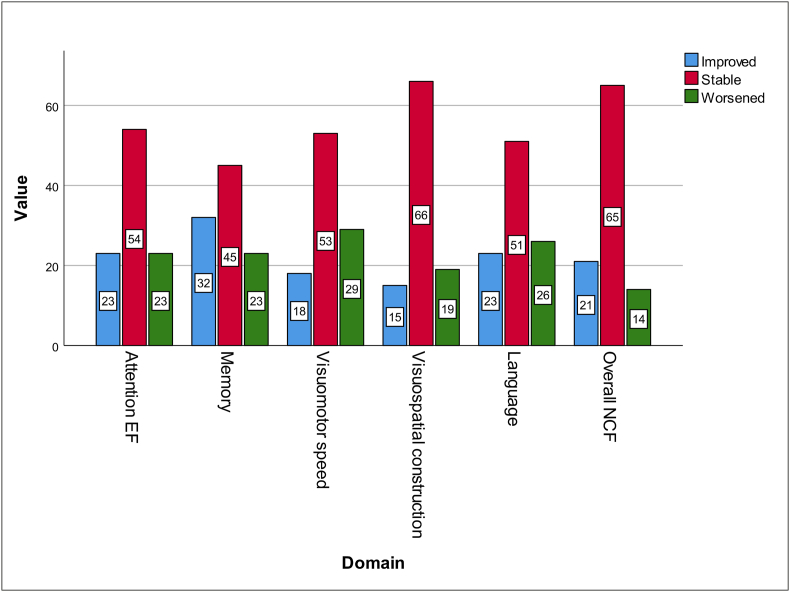
Individual level postoperative changes for each domain. The blue bars on the left indicate improvement, the blue bars in the middle represent stable performance and the green ones on the right show worsening.

Assessment of pre and postoperative severe deficits at the group level or in terms of the percentage of subjects with deficits can mask individual changes (including improvements) in domain functions in the patients. Hence, the percentage of new onset worsening and improvement was also evaluated (Fig. 3). This revealed that Memory showed the maximum improvement (32 %) rate resulting in net improvement as reflected in the overall deficits assessment at the group and individual levels. The improvement rates for A-EF and Language were 23 % each. Visuospatial construction showed the least improvement at 15 %, but it also had the lowest worsening rates (19 %). Visuomotor speed showed the highest worsening rate at 29 %.

**Fig. 4:**
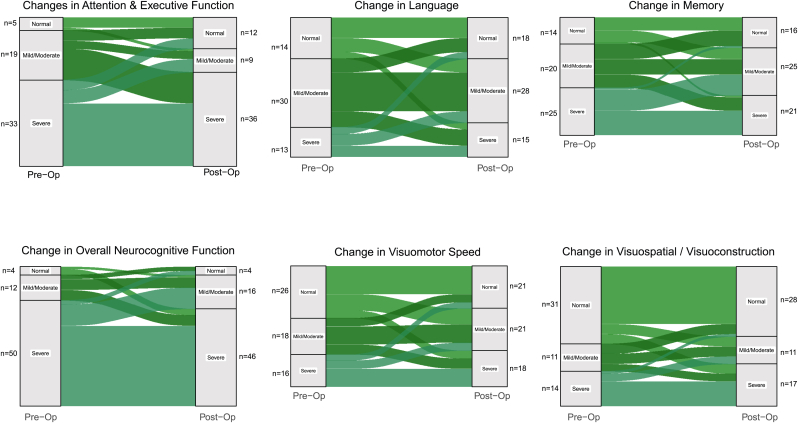
River plots for individual domain as well as overall neurocognitive function. The dynamic changes (improvements as well as worsening) can be very well appreciated across all domains.).

River plots reveal the dynamic and complex flux occurring postoperatively (Fig. 4).

### 3.3 Factors affecting postoperative NCF outcomes

At the group performance level (median scores), no particular factor was significant for overall NCF. Factors observed to be significant for worsening rate are given in Table 3. Extent of resection did not influence the outcomes except for visuomotor speed, which was found to have worsened (p = 0.04) in the non-GTR group in comparison to the GTR group. Right-sided tumours showed a significant worsening in the visuospatial domain (p = 0.04). No significant differences were found in the performance of subjects with low-grade gliomas (LGGs) as compared to those with glioblastomas (GBMs). Tumours smaller than 4 cm tended to show less worsening in visuospatial ability, visuomotor speed and language domains, but this was not seen to be statistically significant. Cognitive reserve factors like residence type, education and occupation were not found to be significantly associated with performance on any domain as well as overall NCF (data not shown). Significant worsening in memory (p = 0.02) was seen in patients who underwent surgeries under GA than those who underwent AC. Worsening was also more for Attention and EF in the GA group, but this was not statistically significant. Table 4 depicts the domain-wise worsening/improvement rates at an individual level for GA and AC groups comparatively.

**Table 3:** Factors affecting worsening of NCF at individual level (percent worsening of patients).

		Attention EF	Memory	Visuomotor	Visuospatial	Language	Overall
Surgery	AC	13.1	21.9	18	17.1	22	13.5
	GA	42.1	52.4	19	9.5	25	36.2
	p value	0.32	**0.02**	0.98	0.50	0.44	0.71
Laterality	Left	21.9	23.5	35.3	9.7	28.6	21
	Right	24	21.4	19.2	32	23.1	3.6
	p value	0.85	0.84	0.17	**0.04**	0.63	0.67
Histology	LGG	22	24.4	26.2	26.3	26.7	14.6
	GBM	28.6	20	37.5	6.25	28.6	12.5
	p value	0.71	1	0.52	0.14	1	1
EOR	GTR	18.5	25	17.6	20	26.5	8.6
	Non GTR	26.67	20	40	19.2	26	19.4
	p value	0.46	0.64	**0.04**	0.94	0.96	0.28
Tumour size	>4 cm	26.3	21	36.8	31.6	31.6	10
	<4 cm	21.05	21.4	21.4	13.5	22	13
	p value	0.74	1	0.32	0.16	0.52	1

LGG - lower grade gliomas; GBM -glioblastoma; GTR - gross total resection.

**Table 4:** Distribution of patients’ post-op status in the awake (AC) vs general anaesthesia (GA) groups (All values in percentage).

	Improvement		Stable		Worsening	
Domain	AC	GA	AC	GA	AC	GA
Attention & EF	18.4	31.6	68.5	26.3	13.1	42.1
Memory*	31.8	4.8	46.3	42.8	21.9	52.4
Visuomotor speed	28.2	28.6	53.8	52.4	18	19
Visuospatial construction	22.9	14.3	60	76.2	17.1	9.5
Language	29.3	20	48.7	55	22	25
Overall	16	9.2	70.5	54.6	13.5	36.2

*p = 0.022, statistically significant.

## 4 Discussion

A high prevalence of baseline neurocognitive deficits in Indian patients with brain tumours has been reported previously highlighting deficits in multiple domains (Moiyadi et al., 2023). Previous studies across the world have reported that a significant number of patients also develop post-operative cognitive deficits (Zetterling et al., 2020; van Kessel et al., 2020; Yordanova et al., 2011; Zarino et al., 2020). This postoperative worsening could be due to variables like oedema, post-operative seizures or the proximity of the resection site to eloquent areas (Zetterling et al., 2020; van Kessel et al., 2020). Literature suggests that most patients have a “V-shaped” trajectory for cognitive performance with baseline impairments followed by transient postoperative worsening in the immediate short-term which typically resolve within weeks (Rijnen et al., 2019; Nakajima et al., 2019; De Witt Hamer et al., 2012; Noll et al., 2024). However, most patients need adjuvant treatments during this period which may itself have adverse effects on cognition in the longer term due to neurotoxicity (Kirkman et al., 2022; van Kessel et al., 2020; Noll et al., 2024). To understand the impact of surgery alone, neurocognition prior to commencing any other oncological treatment needs to be assessed. Studies point to certain factors such as preoperative neurological status (Zarino et al., 2020), the impact of neurosurgical intervention (Kirkman et al., 2022; Yordanova et al., 2011; Lemaitre et al., 2022), tumour characteristics (Kirkman et al., 2022) and functional brain changes (Yordanova et al., 2011) which influence the neurocognitive status dynamically. In the present study, we tried to study factors affecting neurocognitive function in the perioperative period in an Indian cohort.

### 4.1 Post-operative dynamic flux and severity shift

**Fig. 5:**
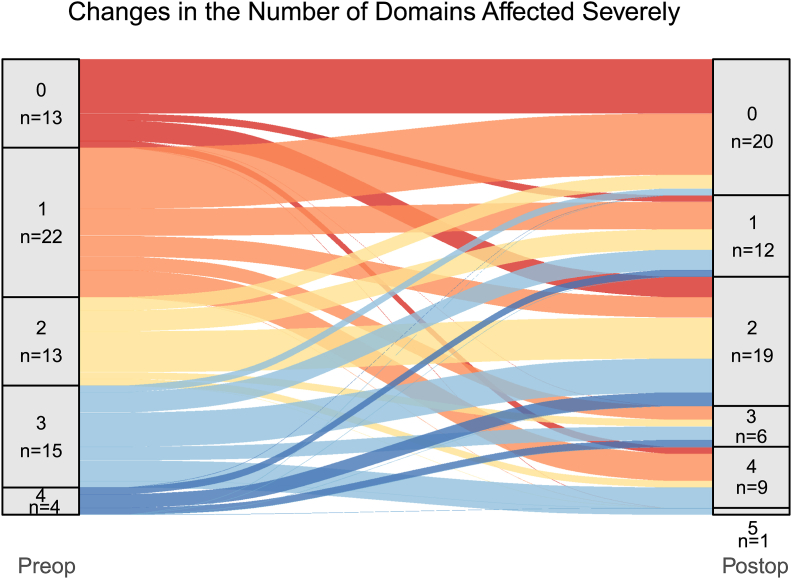
River plot showing severely affected patients pre and postoperatively as per number of domains affected.

We had a high level of baseline dysfunction (almost 76 %), Baseline neurocognitive dysfunction is an important predictor of postoperative outcome (Zangrossi et al., 2022). At the same time, very high prevalence of overall NCF dysfunction at baseline to begin with could mask changes at the domain level and give a false appearance that most patients remained unchanged in NCF, as was seen in our cohort (floor effect). Postoperatively, even when the proportion of patients with severe deficits increased across most domains (except memory), the proportion of patients with severe deficits in the overall NCF decreased. That is because there was a “severity shift” with more patients variably migrating to “mild-moderate” and “severe” group as is evident in the river plots (Fig. 4, Fig. 5), the shifts being variable across domains, as well as transition from single domain to multidomain deficits. Further, both improvement and worsening in different domains could occur in the same patient, and merely assessing the overall NCF (and not a specific domain) using a screening tool or using group performance (as opposed to individual changes) can mask the true nature of changes seen. This has been highlighted earlier by other authors, too (8,10, 11, 12). Hence, assessing individual domains is important to understand the changing pattern of NCF perioperatively, as seen in river plots (Fig. 4).

### 4.2 Influences of awake craniotomy on neurocognitive outcomes

A large proportion of our patients experienced varying degrees of postoperative worsening. Of note is the better performance of patients undergoing AC. In our experience, we observed less decline in post-op NCF in AC over the GA group. This was significant for memory domain and less so for the other domains. Awake craniotomies are undertaken with the intent to preserve NCF. At our centre, we employ tests specific to the domain at risk on the basis of tumour location and involvement of white matter fibres (including tasks of various language components, working memory and executive functions). It appears that memory was benefitted from this mapping process. In the short term, there is still some worsening in NCF, even in patients undergoing AC (Rijnen et al., 2019). We know that in AC with positive mapping, these deficits seen in the immediate post-operative period (van Kessel et al., 2020) recover over time (De Witt Hamer et al., 2012; Yordanova et al., 2011; Zarino et al., 2020). The decline in itself might signify the impact of the process of surgical resection on neurocognition, and AC could have potentially mitigated the impact of this decline, which may otherwise have been more severe. However, we must acknowledge the magnitude of this decline and ensure appropriate acute rehabilitation for these patients in order to optimize their recovery over time. Further refining and adapting intraoperative mapping and monitoring as per baseline NCF status is crucial to improve postoperative outcomes. A longer follow-up would clarify if these patients recovered from the post-operative decline and restore their cognitive functioning to the pre-operative baseline. This was best illustrated by language deficits in our cohort. Language is also the most frequently and extensively mapped function during AC, and it is well known that when positively mapped intraoperatively, the incidence of post-op deficits is very high (Zetterling et al., 2020; De Witt Hamer et al., 2012). The correlation between these outcomes and intraoperative mapping findings is however beyond the scope of the study. It has been observed by many studies that most of these deficits recover in about a few weeks to 3 months (De Witt Hamer et al., 2012; Noll et al., 2024). Our sample also had more cases with left-sided tumours, which is known to be dominant for language and could account for the postoperative language worsening overall. On the other hand, language as a domain showed the second-highest improvement rate. Indeed, AC cases showed higher improvement rates and marginally lower worsening rates in comparison to the GA group.

### 4.3 Interrelationships between domain flux, type of surgery and EOR

Memory domain had the highest improvement rates, especially for those undergoing AC, consequently showing a reduction in the number of overall patients with severe deficits. Relief of mass effect could be one explanation for this observation (Lemaitre et al., 2022). There were significantly more post-operative memory deficits in the GA group compared to the AC group. A trend towards more worsening was seen for those who underwent surgery under GA in comparison to AC across all domains (Noll et al., 2024). Also, interestingly, outcomes were not worse in the radically resected cases (GTR did not adversely influence NCF outcomes). It is pertinent to note that the better neuropsychological performance postoperatively was in spite of much higher GTR rates in the AC group. Indeed, the GTR itself was not associated with adverse outcomes in our cohort, and in fact could have facilitated improvements. This underlines the value of AC in improving not only radicality of resections, but also NCF outcomes, even in so called eloquent regions (Nakajima et al., 2019; Lemaitre et al., 2022).

## 5 Limitations and further directions

The sample size of our study was relatively small to establish causal relationships between the factors studied and neurocognition outcomes, though it did reveal trends in factors like the extent of resection, tumour laterality and type of surgery (AC). This reinforces the need for systematic serial assessments of NCF. Whereas preoperative NCF assessment can help plan surgery in case of awake craniotomies and serve as a metric of disease severity, post-operative scores can potentially provide an insight into the patient outcomes following surgical intervention and, more importantly, improve patient care by informing rehabilitation strategies. Understanding the impact of certain factors on specific domains can help optimize protocols for surgery in order to minimize postoperative sequelae. Basing severity ratings on the lowest score observed and classifying a cognitive domain as impaired if any one of the corresponding tests had an impaired performance was done to detect subtle deficits and ensure high sensitivity. There is a trade-off between sensitivity and specificity. While this approach may disregard intra-individual variability, our objective was to detect the prevalence of dysfunction, and higher sensitivity was deemed to be more appropriate. We also acknowledge the lack of control data in this study, though normative data was used to calculate z scores for tests where this was available. Another potential limitation of the study could be the skewed representation of lower grade gliomas. Being a specialised neurooncological centre, we have a preponderance of patients with DLGGs who are referred for awake craniotomy. Also, patients with higher grade tumours such as glioblastomas are often seen to have acute symptoms characterized by very low KPS and severe cognitive dysfunction; and are unable to undergo a comprehensive neurocognitive assessment. Beyond immediate postoperative outcomes, a long-term follow-up will help reveal patient outcomes over time. Longitudinal time-series NCF data can also be useful in predicting disease progression, progression-free survival and overall quality of life. It is, therefore, important to incorporate neurocognitive evaluation into the routine standard of care in neuro-oncology.

## 6 Conclusions

NCF is a complex multi-domain function. Patients with brain tumours present with a significant burden of NCF deficits at baseline, which may further be compromised after surgery. AC can mitigate the effect of this decline. Careful and serial evaluation of NCF is essential to understand its evolving status over the course of treatment and allows tailored rehabilitative strategies for these patients.

## Funding information

The authors would like to acknowledge the Department of Biotechnology (10.13039/501100001407DBT) for funding the senior author (AM) [Grant - BT/PR42816/MED/122/299/2021].

## Declaration of competing interest

The authors declare the following financial interests/personal relationships which may be considered as potential competing interests:Aliasgar Moiyadi reports financial support was provided by Departement of Biotechnology, Govt of India. If there are other authors, they declare that they have no known competing financial interests or personal relationships that could have appeared to influence the work reported in this paper.
